# A Novel Colorimetric Nano Aptasensor for Ultrasensitive Detection of Aflatoxin B1 Based on the Exonuclease III-Assisted Signal Amplification Approach

**DOI:** 10.3390/foods10112568

**Published:** 2021-10-25

**Authors:** Yu Chen, Fuyuan Zhang, Ruobing Liu, Minxuan Liu, Yaxin Sang, Shuo Wang, Xianghong Wang

**Affiliations:** 1 College of Food Science and Technology, Hebei Agricultural University, Baoding 071001, China; chenyu202100@163.com (Y.C.); zhang.fuyuan@hotmail.com (F.Z.); Koori0520@163.com (R.L.); lmx0325@126.com (M.L.); sangyaxin@hebau.edu.cn (Y.S.); 2 Medical College, Nankai University, Tianjin 300500, China

**Keywords:** aflatoxin B1, aptamer, G-quadruplex, Exonuclease III, signal amplification

## Abstract

The detection of aflatoxin B1 (AFB1) has recently garnered much attention on the issue of food safety. In this study, a novel and sensitive aptasensor towards AFB1 is proposed using an Exonuclease III (Exo III)-integrated signal amplification strategy. This reported sensing strategy is regulated by aptamer-functionalized nanobeads that can target AFB1; furthermore, complementary DNA (cDNA) strands can lock the immobilized aptamer strands, preventing the signal amplification function of Exo III in the absence of AFB1. The presence of AFB1 triggers the displacement of cDNA, which will then activate the Exo III-integrated signal amplification procedure, resulting in the generation of a guanine (G)-rich sequence to form a G-4/hemin DNAzyme, which can catalyze the substrate of ABTS to produce a green color. Using this method, a practical detection limit of 0.0032 ng/mL and a dynamic range of detection from 0.0032 to 50 ng/mL were obtained. Additionally, the practical application of the established sensing method for AFB1 in complex matrices was demonstrated through recovery experiments. The recovery rate and relative standard deviations (RSD) in three kinds of cereal samples ranged from 93.83% to 111.58%, and 0.82% to 7.20%, respectively, which were comparable with or better than previously reported methods.

## 1. Introduction

Aflatoxin B1 (AFB1) produced by the Aspergillus species is one of the most toxic mycotoxins occurring in agricultural products, and it can directly contaminate cereals like corn, wheat, barley, millet, and their by-products [1,2,3]. Exposure to this toxic substance can lead to serious consequences for human health, including hepatotoxicity, carcinogenicity, teratogenicity, and mutagenicity [4,5]. For this reason, many countries and organizations have established maximum residue limits (MRLs) of AFB1 in cereal foodstuffs to ensure food safety. For example, the U.S. FDA established 20 μg/kg as the MRLs of AFB1 in most of that country’s cereals [6].

Up to now, numerous analytical techniques have been employed for quantitative determination of AFB1, such as thin layer chromatography (TLC) [7], high performance liquid chromatography (HPLC) [8], and liquid chromatography-mass spectrometry (LC-MS) [9]. These instrumental analytical methods have high sensitivity, good repeatability, and strong stability for AFB1 detection. However, they constantly require expensive instruments and highly trained personnel, and the complex sample processing stage is cumbersome and time-consuming. Issues such as these restrict their applications in the economical and easy-to-use monitoring of AFB1 [10].

Nucleic acid aptamer, which is referred to as a “chemical antibody”, has been widely used as a replacement of the traditional antibody in analytical chemistry with some remarkable advantages over the antibody [11,12]. For example, these include higher chemical stability, cost-effective production and extended storage, and the fact that the aptamer can be artificially synthesized in vitro with a similar binding affinity to the antigen-antibody interaction [13]. Many biosensors for AFB1 have been devised using antibody-mimetic aptamers. One of the most important features of the aptamer is that it can be easily designed and modified for introducing signal amplification strategies to improve the method’s detection sensitivity [14]. Exonuclease III (Exo III) has been employed as an enzyme-catalyzed target-recycling signal amplification for ultrasensitive target detection because the signaling probes can be selectively digested by Exo III with a target DNA cycling [15,16]. It is able to produce a superimposed concentration of a certain signal DNA to finally achieve signal amplification [17]. Exo III-based methods are sensitive and selective for the detection of targets in appropriate conditions because these methods have excellent characteristics [18]. Some studies have designed the produced signal DNA as a guanine (G)-rich sequence strand which can be folded into a highly ordered G-quadruplex (G-4) structure through the hydrogen bonding of Hoogsteen base pairs between guanine G and the interaction between cations and quadruplex structures [19,20,21]. The stable G-4 can be combined with hemin to catalyze H_2_O_2_ and 2,2′-diazo-bis (3-ethyl-Benzothiazoline-6-sulfonic acid) diamine salt (ABTS) so that it changes from colorless to green [22,23]. Based on this principle, signal amplification and the detection of metal ions [24,25], DNA [26], organic molecules, and proteins are possible [27].

Herein, we designed a novel Exo III-assisted signal amplification strategy for colorimetric sensing of aflatoxin B1. A nano aptasenor was firstly established by modifying AFB1-specific aptamers on the surface of magnetic nanobeads, and then the binding site of the aptamer strand was blocked through the hybridization with its complementary DNA (cDNA). In the signal amplification procedure, a G-rich strand DNA (S2) and its complementary strand DNA (S1) were designed to form a duplex DNA with 3′ overhangs containing more than 4 bases, which inhibits the catalytic activity of Exo III. The target AFB1 molecule can trigger the release of cDNA due to the specific interaction between AFB1 and the aptamer strand. The released cDNA can hybridize with the S1–S2 double-strand DNA to form a blunt end which will be degraded by Exo III from the 3′-end of the S1 strand. The released cDNA can trigger another Exo III-induced signal amplification process, while the S2 strand can form a G-4 structure DNAzyme. In the presence of hemin, the G-4/hemin DNAzyme shows peroxidase-mimicking catalase activity which will catalyze the colorless ABTS to form green ABTS^2+^. Based on this, AFB1 could be quantitatively detected by monitoring the increase of the absorbance value. In this study, the developed sensor was successfully employed to detect AFB1 in three kinds of cereal samples. We found that the detection limit of the amplification procedure-introduced sensing strategy has high selectivity, high sensitivity and low cost, and furthermore does not require any complex chemical modification.

## 2. Materials and Methods

### 2.1. Reagents and Standards

Standards of AFB1, AFM1, ochratoxin A (OTA), fumonisin B1 (FB1), T2 toxin (T2), deoxynivalenol (DON), and zearalenone (ZEN) were purchased from Pribolab Co., Ltd. (Shenzhen, China). The TE buffer was purchased from Solarbio Co., Ltd. (Beijing, China). The 2, 2′-azino-bis (3-ethylbenzothiazoline-6-sulfonic acid) diamine salt (ABTS) was purchased from Meryer Co., Ltd. (Shanghai, China). Hemin was obtained from Sigma Co., Ltd. (Saint Louis, MI, USA). Streptavidin immobilized magnetic beads were purchased from Beaverbio Co., Ltd. (Jiangsu, China), while the commercial ELISA kit for AFB1 was bought from MEIKE Co., Ltd. (Jiangsu, China). Other reagents used in this experiment were sourced from Sinopharm Chemical Regent Co., Ltd. (Shanghai, China). The working buffer, consisted of 10 mM Tris-HCL buffer that contained 50 mM NaCl, 10 mM MgCl_2_, and 100 mM KCl, PH 8.0. The ABTS incubation buffer consisted of 5 mM Na_2_HPO_4_·12H_2_O and 5 mM C_6_H_8_O_7_·H_2_O, pH 3.6, and stored at 4 °C. The ABTS working solution contained 100 μL of 72 mM ABTS, 10 μL 30% H_2_O_2_, and 890 μL ABTS incubation buffer.

Lastly, the AFB1 aptamer, cDNA, S1 and S2 oligonucleotides were synthesized by Sangon Biotechnology Co., Ltd. (Shanghai, China). The sequences of the oligonucleotides are as follows:

FAM-labeled AFB1 aptamer: 5′-FAM-TGC ACG TGT TGT CTC TCT GTG TCT CGT GCT TTT TT-3;

BHQ1-labeled cDNA: 5′-AGA CAA CAC GTG CAA TAA C-BHQ1-3′;

AFB1 aptamer: 5′- TGC ACG TGT TGT CTC TCT GTG TCT CGT GCT TTT TT-Biotin-3′;

cDNA: 5′-AGA CAA CAC GTG CAA TAA C-3′;

S1: 5′-AAA ACC CAA AAC CCA AAA CCC TGC ACG TGT TGT CT-3′;

S2: 5′-CAC ATG CTG GGT TTT GGG TTT TGG GTT TTG GGA GCT A-3′.

### 2.2. Instrumentation

Fluorescence spectra and fluorescence intensity were measured by a F-320 fluorescence spectrophotometer manufactured by Gangdong Sci. & Tech. Co., Ltd. (Tianjin, China). The emission spectrum was recorded in the 515–700 nm wavelength range under the excitation at 492 nm, while the slit width was set to 10 nm. A microplate reader from Thermo Scientific Co., Ltd. (Shanghai, China) functioned to read the colorimetric value at 415 nm. A metal bath in the experiment was obtained from Shanghai Longyue Instrument Equipment Co., Ltd.

### 2.3. Optimization of AFB1-Induced cDNA Displacement from Aptamer

FAM-labeled aptamer (50 μL, 500 nM) was injected in a 200 μL tube, and then 50 μL of different concentrations of BHQ1-labeled cDNA were added (0 nM, 125 nM, 250 nM, 500 nM, 1000 nM, and 1500 nM). Then the tubes were placed in the metal bath and incubated at 37 °C for about 30 min. The fluorescence intensity at 525 nm was recorded with the excitation at 492 nm. The hybridization ratio of the aptamer and cDNA was optimized.

For the optimization of hybridization time between aptamer and cDNA, FAM-labeled aptamer (50 μL, 500 nM) and BHQ1-labeled cDNA (50 μL, 500 nM) was first mixed adequately, and then incubated at 37 °C for 3 min, 5 min, 10 min, 15 min, 20 min, and 30 min, respectively. The fluorescence intensity at 525 nm was recorded with the excitation at 492 nm.

Lastly, the reaction time between the aptamer–cDNA and AFB1 was optimized. The FAM-labeled aptamer (50 μL, 500 nM) and BHQ1-labeled cDNA (50 μL, 500 nM) were first mixed adequately, and then 50 μL of 2 ng/mL AFB1 was added and incubated at 37 °C for 10 min, 20 min, 30 min, 40 min, 50 min, and 60 min, respectively. Fluorescence intensity at 525 nm was recorded with the excitation at 492 nm.

### 2.4. Optimization of G-4/Hemin Colorimetric Conditions

For optimizing the hemin concentration, 20 μL of 1 μM S2 was put into a 96-well microtiter plate and 35 μL of working buffer was added to it. Then, 60 μL of different concentrations of hemin (0, 0.5, 1, 2, 4, 8, 16, and 32 μM) were added, and they were put into an incubator at 37 °C for 10 min. Following that, 40 μL of ABTS working solution was added and incubated at 37 °C for 10 min. Finally, the absorbance value at 415 nm was measured.

For the optimization of the hemin incubation time, 20 μL of 1 μM S2 was put into a 96-well microtiter plate and to it was added 35 μL of working buffer. Then, 60 μL of 4 μM hemin was added to assess the reaction in a 37 °C incubator for different amounts of time (0, 5, 10, 15, 20, 25, and 30 min). After that, 40 μL of ABTS working solution was added to see the reaction in a 37 °C incubator for 10 min. Lastly, the absorbance value at 415 nm was measured.

For the optimization of the colorimetric temperature, 20 μL of 1 μM S2 was put into a 96-well microtiter plate and to it was added 35 μL of working buffer. Following this, 60 μL of 4 μM hemin was added to see the reaction for 10 min at temperatures of 4 °C, 25 °C, 37 °C, and 50 °C, respectively. Then, 40 μL of ABTS working solution was added and incubated at 37 °C for 10 min. Completing this process was measuring the absorbance value at 415 nm.

### 2.5. Detection of AFB1 Using the Developed Aptasensor

The 60 μL of streptavidin immobilized magnetic beads (1 mg/mL) were put into a 1.5 mL tube, and then washed twice with 200 μL of working buffer after the magnetic separation. Next, 100 μL aptamer (200 nM) was situated in the washed beads to test the reaction for 30 min in a shaker (150 rmp) at 37 °C. The beads were subsequently washed twice with 200 μL of working buffer in a magnetic field, followed by adding 100 μL of AFB1 with different concentrations (0, 0.0032, 0.016, 0.08, 0.4, 2, 10, 50 ng/mL) in a metal bath for 20 min under 37 °C for reaction purposes. In the next step, 100 μL of cDNA (200 nM) was added after being twice washed with 200 μL of working buffer. The mixture was placed in a metal bath at 37 °C for 3 min, and then the tubes were positioned in a magnetic field. Then 10 μL of supernatant was put into 40 μL of a pre-processed S1-S2 duplex solution. It should be noted that the 1 μM of S1 and 1 μM of S2 were equally mixed and pretreated at 95 °C for 3 min, and then cooled to room temperature naturally. With this done, 5 μL of Exo III (10 U/μL) was added for incubation lasting 1 h at 37 °C. After that, 60 μL of hemin (4 μM) was added and incubated for 10 min at 37 °C. Finally, 40 μL of ABTS working solution was added and subjected to a reaction for 8 min in an incubator at 37 °C. The absorbance value at 415 nm was recorded using a microplate reader.

### 2.6. Treatment of the Real Cereal Samples

Maize, millet, and sorghum were first ground and filtered with a mesh size of 2 mm. Next, varying concentrations of AFB1-spiked samples (0.5 g) were extracted using 2 mL methanol–water mixture (70:30, *v*/*v*) and shook continuously for 10 min. After centrifugation for 10 min (6000 rmp/min), the supernatant and dilute were taken 5 times to obtain the sample extract that would be used for testing.

## 3. Results and Discussion

### 3.1. Principle of the Signal Amplification-Based Aptasensor

**Figure 1:**
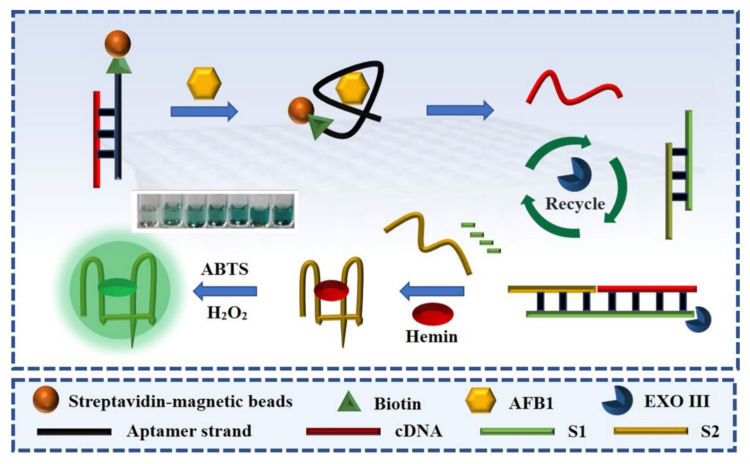
Schematic illustration of the Exo III-induced signal amplification aptasensor for the colorimetric detection of aflatoxin B1 (AFB1).

As shown in Figure 1, in this amplification reaction system, the designed DNA probes S1 and S2 can be hybridized to form a stable toehold duplex structure (S1/S2), and in this case, the quadruplex-forming oligomer (S2) strand will not form a stable G-quadruplex structure. In the presence of AFB1, the aptamer immobilized on the magnetic nanobead opens to combine with the target AFB1, inducing the conformational change of the aptamer and releases its complementary primer chain (cDNA) from the magnetic nanobeads. The cDNA is the starting strand that triggers the amplification cycle. When the duplex probe (S1/S2) is challenged with the released cDNA, the primer cDNA can hybridize with its complementary sequence parts in the duplex probe (S1/S2), and the hybridization leads to a blunt 3′-terminus, which can be catalyzed by Exo III. Subsequently, the cDNA and a quadruplex-forming oligomer (G-4) can be released. The intriguing interaction between the G-4 structure and its specific ligand, hemin, brings about a peroxidase-like activity to catalyze the colorless ABTS^2-^ into green ABTS^2+^ under the influence of H_2_O_2_. During the Exo III catalysis stage, the retrieved cDNA is ready to bind to another S1/S2 probe to activate a new cycle, generating a concomitant increase in the catalysis ability of the G-4 DNA enzyme.

### 3.2. Optimization of AFB1-Induced cDNA Displacement from Aptamer

The AFB1-induced displacement of the cDNA from the aptamer is the key not only for the recognition unit of the target but also for the starting unit of the amplification reaction. We tested the feasibility of this process for AFB1 detection employing a FRET analysis [28,29]. We labeled the anti-AFB1 aptamer and its complementary DNA with a fluorescence probe (FAM) and quencher probe (BHQ1), respectively. When AFB1 was not present, the FAM-labeled aptamer hybridized with the BHQ1-labeled cDNA, generating a decreased fluorescence signal. When AFB1 existed in the buffer solution, the signal significantly increased, which confirmed the displacement of the cDNA from the aptamer. To design the truncated cDNA probe, we divided the structure of the reference aptamer into two halves based on previous reports describing AFB1-aptamer kissing complexes [30]. Furthermore, we assumed that the first part of the aptamer contained 14 nucleotides from the 5′end (1 to 14) and might form a kissing complex and facilitate AFB1-aptamer complex formation. Thus, its complementary cDNA (14 nucleotides-length) was designed by using UNAFold for the competitive displacement assay.

**Figure 2:**
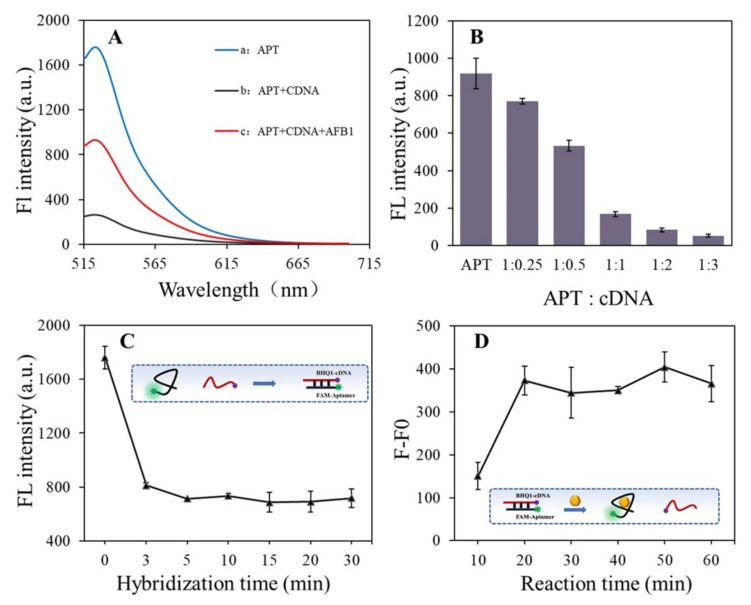
(**A**) Fluorescence effect of FAM-aptamer and BHQ1-cDNA in the structure-switch displacement assays. (**B**) Optimization of the hybridization ratio of aptamer and cDNA. (**C**) Optimization of the hybridization time of aptamer and cDNA. (**D**) Optimization of the reaction time between the aptamer–cDNA and AFB1. F and F0 are respectively the fluorescence intensity in the presence and absence of AFB1.

As can be seen from Figure 2A, fluorescence intensity of the FAM-aptamer in the duplex dropped to 85% due to FAM-BHQ1 pairing. When 2 ng/mL AFB1 existed in the solution, there was a significant increase in the fluorescence intensity of the aptamer–cDNA duplexes (about 72%), which revealed the formation of the aptamer-AFB1 complex and subsequent dissociation of BHQ1-labeled complementary cDNA. The fluorescence for switching in the duplex was monitored by increasing this fluorescence intensity, which confirmed that separation of the aptamer–cDNA pair did occur.

In order to obtain better cDNA displacement efficiency, the hybridization ratio of the aptamer and cDNA was first optimized. As shown in Figure 2B, the fluorescence of the aptamer–cDNA duplex decreased when the hybridization ratio (aptamer: cDNA) changed from 1:0.25 to 1:3, which indicated that the formation of the duplex specifically depended on the concentration of the cDNA probe. When the hybridization ratio reached 1:1, the FAM fluorescence quenching efficiency achieved virtually the same. Although higher quenching efficiency generated for the duplex contains a larger concentration of cDNA (1:2 and 1:3), the hybridization ratio of 1:1 was selected for the following experiment. It took into account that the excess of cDNA may cause a higher signal background in the cDNA-induced signal amplification process.

Figure 2C presents the ideal time required for the aptamer–cDNA hybridization when the ratio of cDNA/aptamer was set as 1:1, determined in the single-variable experiment. After 3 min, the reduced value of the fluorescence signal slows down, which strongly suggested that the free aptamer strands and the cDNA strands in the solution almost form the aptamer–cDNA duplexes in a short time, and induce the FRET between the FAM and BHQ1. On this basis the aptamer–cDNA hybridization time was determined to be 3 min. In addition, the aptamer is the key as the recognition unit of the target. Thus, the incubation time between the aptamer and AFB1 was greatly improved (Figure 2D). As time passed from 10 min to 60 min, the fluorescence recovery (F–F_0_) gradually increased. After 20 min, the fluorescence recovery value tended to be stable although it fluctuated, which revealed that most of the AFB1-aptamer recognizing and binding process could finish within 20 min. Therefore, the AFB1-aptamer reaction time of 20 min was chosen to ensure that the sufficient combination of aptamer and AFB1 was realized. In the optimized scenario, 2 ng/mL of AFB1 can induce about 4 times the signal recovery in the aptamer–cDNA duplex system.

This section may be divided by subheadings. It should provide a concise and precise description of the experimental results, their interpretation, as well as the experimental conclusions that can be drawn.

### 3.3. Optimization of G-4/Hemin Colorimetric Conditions

**Figure 3:**
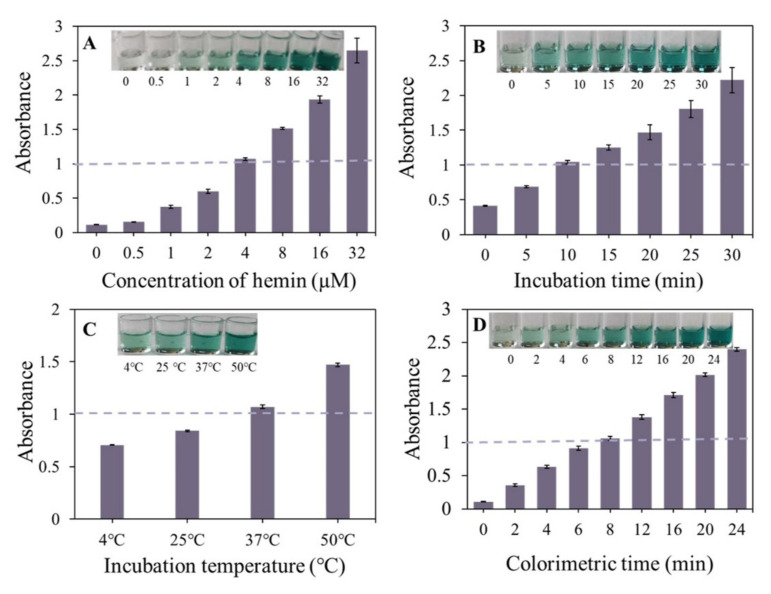
Optimization of the G-4/hemin colorimetric conditions. (**A**) Concentration of hemin. (**B**) Incubation time of between G-4 and hemin. (**C**) Working temperature. (**D**) Reaction time between G-4/hemin and ABTS.

The G-rich sequences associate with hemin to form massive peroxidase-mimicking DNAzymes [31], which can catalyze the oxidation of the colorless ABTS^2−^ to green-colored ABTS^2+^ in the presence of H_2_O_2_. The concentration of hemin, the G-4/hemin incubation time, and the incubation temperature wielded great influence on the formation of the G-4/hemin complex, thereby affecting the catalytic oxidation of ABTS. We firstly studied the colorimetric effect of the hemin concentration in the presence of 1 μM G-4 strand (S2), finding that a small colorimetric response (background) was obtained for the hemin probe free solution. In addition, the absorbance value of the G-4/hemin colorimetric system at 415 nm increased upon increasing the concentration of hemin (from 0 to 32 μM) in the 0.11–2.65 a.u. range (Figure 3A). According to Figure 3B, the absorbance value also gradually rises along with the increasing interaction time between the hemin and the G-4 from 0 to 30 min. In addition, it was found here that the absorbance value increased when the experiment working temperature was ramped up (Figure 3C). Based on the above optimization, the hemin concentration, incubation time, and working temperature of the G-4/hemin colorimetric system were set to 4 μM, 10 min, and 37 °C, respectively, to obtain a desirable optical density value of 1 a.u. After adding ABTS in the G-4/hemin, the absorbance values increased rapidly, and it only needed 8 min to reach 1 a.u. (Figure 3D), which indicated that the G-4/hemin has a high catalytic activity towards ABTS. For this reason, the explicit catalytic time of 8 min was selected as being ideal for colorimetric analysis.

### 3.4. Verification of the Exo III-Induced Signal Amplification Procedure

**Figure 4:**
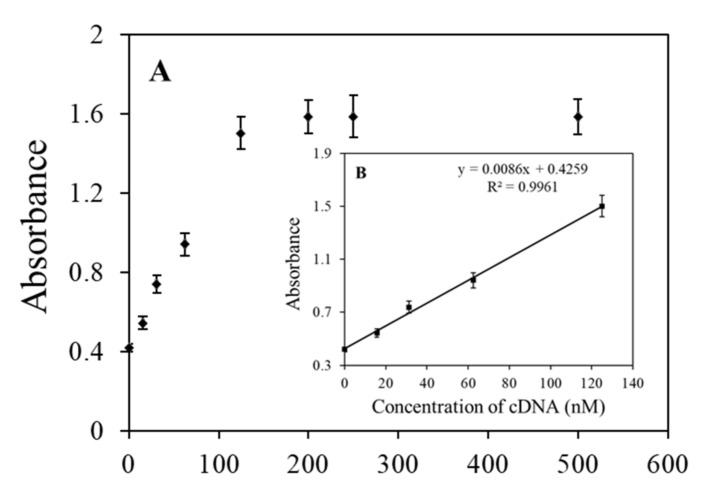
Colorimetric signal changes in the G-4/hemin mixture as a function of the concentration of perfectly matched cDNA. (**A**) Calibration curve for determination of the cDNA; (**B**) Linear correlation at a concentration range of 0–125 nM.

We designed a duplex DNA probe with one DNA strand containing a G-4 DNA sequence, and this G-4 sequence will be covered by a complementary sequence in the duplex DNA, which hampers the ability of the G-4 probe to form a G-4 structure. Meanwhile, the duplex DNA probe is constructed as protruding to ensure the probe cannot be digested by Exo III. In the presence of cDNA, the cDNA can hybridize with the ssDNA at the 5′end of the duplex with leads to create a cleavage site. Exo III can then catalyze the stepwise removal of mononucleotides from this 5′ terminus, releasing the cDNA and the G-4 sequence. The released G-4 sequence forms a G-4 structure by self-folding, which binds to hemin to form the DNAzyme. The released cDNA binds to another 3′-end of the duplex to trigger a new hydrolysis reaction, leading to significant amplification of the signal. In order to verify the performance of this cDNA-induced amplification procedure, we challenged it with a series of cDNA concentrations. The colorimetric signal increased with the cDNA concentration up to 500 nM (Figure 4). There was a linear relationship between the signal and the concentration of cDNA in the 0 to 125 nM range and a correlation coefficient of 0.9961 was obtained. This result indicated that the release of the G-4 sequence greatly depended on the concentration of cDNA.

### 3.5. Construction of the Signal Amplification-Based Nanosenosr for AFB1 Detection

A novel nanosensor was constructed by combining the AFB1-induced aptamer-structure switch process and the Exo III-induced colorimetric signal amplification procedure. In order to reduce the detection background caused by the excess of cDNA in the aptamer-structure switch process, we applied the aptamer-immobilized magnetic nanobeads. They served as the sensing platform to remove the aptamer and aptamer-combined cDNA through magnetic separation. It is worth noting that the introduction of nanobeads can improve the modification efficiency and the stability of the sensing.

**Figure 5:**
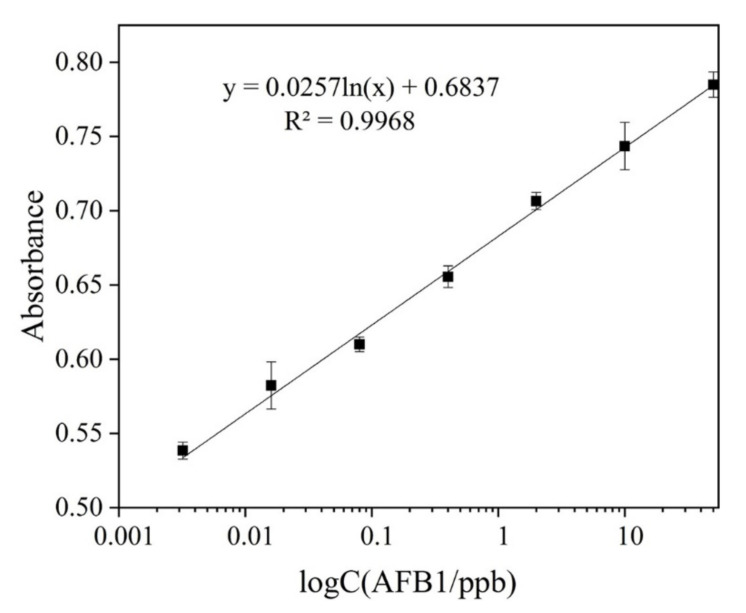
Linear relationship between the colorimetric signal and concentration of AFB1 on logarithmic scales. Each data point represents an average of 3 measurements (each error bar indicates the standard deviation).

Under optimized experimental conditions, the sensitivity of the proposed sensor for AFB1 detection was examined in the working buffer. Figure 5 displays colorimetric signals of the sensing system corresponding to a series of different concentrations of AFB1. As can be seen, the colorimetric signal increases when the concentration of AFB1 is elevated from 0.0032 ng/mL to 50 ng/mL.

By plotting the colorimetric signal vs. the concentration of AFB1, a calibration detection line is obtained for AFB1 detection. The colorimetric signal varies linearly with the AFB1 concentration in the range from 0.0032 ng/mL to 50 ng/mL with a good regression coefficient of 0.9968. The practical detection limit for AFB1 is defined as 0.0032 ng/mL when applying it in the gradient diluted AFB1 solution. Such a detection limit is significantly improved compared to other commonly used approaches or is comparable with many methods coupled with complicated signal amplification strategies for AFB1 detection (Table 1).

**Figure 6:**
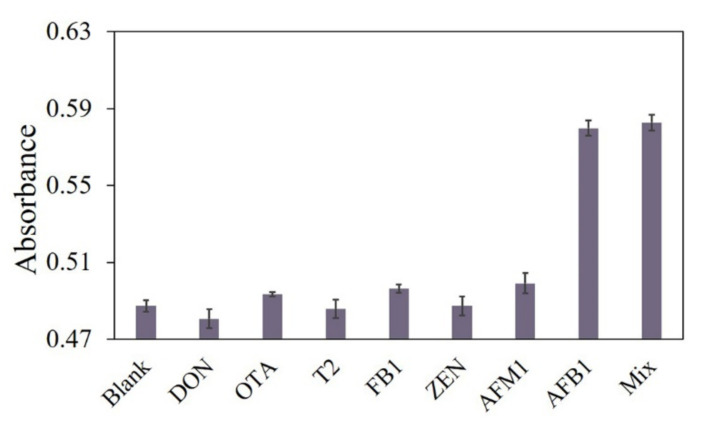
Selectivity investigation of the proposed sensor for DON, OTA, T2, FB1, ZEN, and AFM1 (final concentration 100 ng/mL). The concentration of AFB1 is 10 ng/mL.

To evaluate the selectivity of our proposed aptasensor for the detection of AFB1, monitoring the color change of the solution against other control targets is done by examining DON, OTA, T2, FB1, ZEN, and AFM1 [38]. As shown in Figure 6, the presence of even a 10-fold excess (100 ng/mL) of the control targets causes insignificant color changes in the solutions, while the presence of a smaller concentration of AFB1 (10 ng/mL) results in a substantial increase in the colorimetric signal. More importantly, the mixture of the control molecules and AFB1 wields only a slight influence on the colorimetric signal, revealing that our fabricated colorimetric amplification approach is highly selective for AFB1 detection, and other targets have no interference.

This sensor was designed to detect AFB1 in cereals, and the sample extract may influence the aptamer-AFB1 recognition and signal amplification procedure, and subsequently the guide detection performance. To study the matrix effect, we applied the developed aptasensor in the extract solution and established the extract-calibrated detection curve. As shown in Figure S1, the detection line developed in the extract also demonstrated a high linear relationship. The calibration equation obtained from this curve was y = 0.017lnx + 0.706 with a correlation coefficient of R^2^ = 0.9958. The practical detection limit is also defined to be 0.0032 ng/mL when used in different concentrations of AFB1 in the extract. In order to evaluate the practical application of the proposed aptasensor, we challenged it by using three kinds of cereal samples (10 µg/kg, 20 µg/kg, and 40 µg/kg of AFB1 were spiked to corn samples; and 2.5 µg/kg, 5 µg/kg, and 10 µg/kg of AFB1were spiked to millet and sorghum samples) in identical experimental procedures. The analysis results are summarized in Table 2. The average recovery of the developed sensor ranged from 93.83–111.58%, with the relative standard deviation (SD) varying from 0.82% to 7.20%. The results of the commercial ELISA kit matched well with those obtained from our developed sensor, with a recovery from 83.01% to 94.81%, indicating that the proposed aptasensor can be used as a robust and sensitive detection method for AFB1 in cereal samples.

## 4. Conclusions

In this paper we developed a novel colorimetric nano aptasensor for ultrasensitive detection of AFB1. It is achieved by combining a magnetic nanobeads-based aptamer-structure-switch assay and an Exo III-assisted signal amplification approach. This biosensor demonstrated a higher sensitivity (0.0032 ng/mL) compared with most of the detection methods devised in other research. The practical application of this sensor was also verified through a recovery experiment in three different kinds of cereals by using commercial ELISA kits as a comparison. This ultrasensitive biosensor is a potentially versatile and modular sensing platform for the detection of numerous harmful substances by simply changing other aptamers into molecular recognition probes.

## Figures and Tables

**Table 1:** Comparison of the developed aptasensor with other different detection methods for AFB1 determination.

Methods	Sensing PLATFORM	Detection Range (ng/mL)	LOD (ng/mL)	Reference
Electrochemistry	SPCE/GO-aptamer/MB	0.05–6.0	0.05	[32]
Electrochemistry	Gold electrode/MB/Aptamer	0.6–150	0.6	[33]
Fluorescence	TPE-Z/GO/aptamer	0.25–3	0.25	[34]
Fluorescence	AuNPs-aptamer/QDs	3.1–125	1.06	[35]
Surface-enhanced Raman	Poly (N-acryloyl glycinamide)	10–100	10	[36]
Immunochromatographic	AuNPs/aptamer	0–50	1.05	[37]
Colorimetric assay	Magnetic nanobeads/aptamer	0.0032–50	0.0032	This work

**Table 2:** Applications of the developed sensor for AFB1 in different cereal samples and verification with a commercial ELISA kit. (*n* = 3).

Sample	Spiked AFB1 (µg/kg)	This Work		Commercial ELISA Kits	
		Found (µg/kg)	Recovery (%)	Found (µg/kg)	Recovery (%)
corn	10	11.02 ± 0.72	110.26 ± 7.20	8.30 ± 0.24	83.01 ± 2.37
	20	18.77 ± 0.37	93.83 ± 1.88	17.80 ± 0.31	89.27 ± 1.57
	40	38.27 ± 0.61	95.69 ± 1.53	37.92 ± 1.14	94.81 ± 2.86
millet	2.5	2.36 ± 0.02	94.53 ± 0.82	2.23 ± 0.05	89.18 ± 1.86
	5	4.86 ± 0.17	97.12 ± 3.40	4.68 ± 0.18	93.6 0 ± 3.56
	10	11.16 ± 0.38	111.58 ± 3.79	8.31 ± 0.15	83.0 7 ± 1.47
sorghum	2.5	2.39 ± 0.03	95.65 ± 1.14	2.15 ± 0.03	86.0 3 ± 1.37
	5	5.13 ± 0.10	102.66 ± 2.02	4.34 ± 0.11	86.86 ± 2.24
	10	11.10 ± 0.21	110.96 ± 2.05	9.25 ± 0.25	92.51 ± 2.48

## Supplementary Materials

The following are available online at https://www.mdpi.com/article/10.3390/foods10112568/s1, Figure S1: Extract-calibrated detection curve.
