# Supplementary figures and images for: A Novel Colorimetric Nano Aptasensor for Ultrasensitive Detection of Aflatoxin B1 Based on the Exonuclease III-Assisted Signal Amplification Approach
Source: Foods. 2021 Oct 25;10(11):2568. doi: 10.3390/foods10112568 (PMC8625208; doi:10.3390/foods10112568)

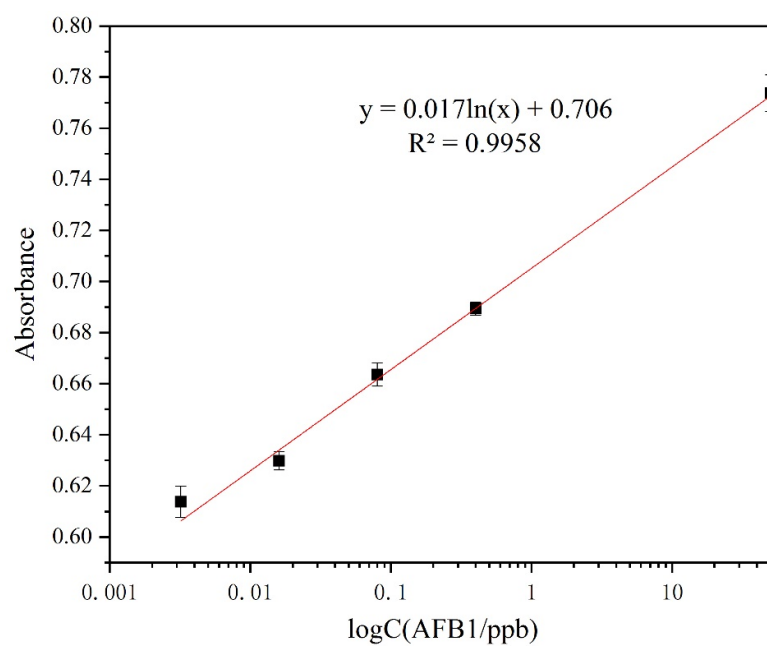

**Figure S1.** Extract-calibrated detection curve.

Supplement: Supplementary file 1 [file foods-10-02568-s001.zip › foods-1420793-supplementary.pdf]
